# De-medicalized and decentralized HIV testing: a strategy to test hard-to-reach men who have sex with men in Cameroon

**DOI:** 10.3389/fpubh.2023.1180813

**Published:** 2023-07-26

**Authors:** Jean Paul Bienvenu Enama Ossomba, Patrice Ngangue, Antoine Silvère Olongo Ekani, Edgar Tanguy Kamgain

**Affiliations:** ^1^School of Health Sciences, University of Dundee, Dundee, United Kingdom; ^2^Faculty of Nursing, Laval University, Québec, QC, Canada; ^3^Humanity First Cameroon Plus, Yaounde, Cameroon

**Keywords:** HIV testing, key population groups, demedicalisation, decentralisation, low-and-middle-income countries

## Abstract

Conventional HIV testing performed by a health professional has shown its limitations in targeting marginalized and vulnerable populations. Indeed, men who have sex with men (MSM) due to social discrimination are often uncomfortable using this service at the health facilities level. In this perspective, new differentiated approaches have been thought through de-medicalized and decentralized HIV testing (DDHT). This HIV testing strategy enables overcoming the structural, legal, and social barriers that prevent these populations from quickly accessing HIV services. This article discusses the prerequisites and added value of implementing this strategy for MSM living in a criminalized context and its implication in decentralizing health services toward the community level.

## 1. Introduction

In 2021, UNAIDS estimated that 38.4 million people were living with HIV worldwide, of whom 5.9 million did not know their HIV status (1). These data are exacerbated in key populations group at higher risk of acquiring this infection (2). Key populations include female sex workers and their clients, gay men and other men who have sex with men, injecting drug users and transgender women.

Epidemiological data from recent studies show that Cameroon faces a mixed HIV/AIDS epidemic (generalized and concentrated). The last Demographic Health Survey (DHS) (3) found a national HIV prevalence of 2.7% in the general population. The IBBS survey conducted among key populations in 2016 found a prevalence of 20.7 among MSM (4). Regarding service provision, data from HIV programs compiled with Spectrum software in 2020 showed that only 506,433 people know their HIV status (5). Therefore, it is imperative to intensify the search for positive cases to reach the first 95 of the UNAIDS 95/95/95 targets (6). For this instance, a change in the traditional paradigm is needed by de-medicalizing the provision of HIV services (7). The new UNAIDS strategy recommends involving the most affected communities at the center of HIV response strategies because, until now, they have always been interested in prevention activities through peer education (8). Data from key populations have shown that peer education facilitates community mobilization of hard-to-reach MSM (9). Hard-to-reach MSMs are those who do not have access to mainstream prevention services. Due to stigma, they rarely go to drop-in centers (DIC) and health facilities (10). In addition, peer education does not always give access to other sexual health services available exclusively in DIC. Hence, the decentralized de-medicalized HIV testing (DDHT) strategy has been developed to provide MSMs with services adapted to their needs. The DDHT allows trained non-healthcare workers to perform rapid HIV testing at the community level while respecting quality assurance and biosafety standards. This paper aims to demonstrate that DDHT is suitable for reaching hard-to-reach MSM in countries where homosexuality is still criminalized. To do so, the structural and legal barriers that MSM face in accessing conventional HIV testing services will be described. The prerequisites for implementing the DDHT and its added value for MSM will be stressed. Finally, the implication of the DDHT strategy in the HIV care and treatment continuum will be shown.

## 2. Methodology

We relied on grey literature data and peer review journal publications to write this article. In the grey literature, we looked at reports and normative documents from UNAIDS, PEPFAR, and the Global Fund to Fight HIV, Tuberculosis and Malaria. We were also interested in national HIV policies focusing on decentralizing HIV services at the community level. Finally, we also took a particular interest in the HIV control programs targeting key populations implemented over the last decade in Cameroon.

Regarding scientific articles, we conducted a literature search on PUBMED, Journal of AIDS (JAIDS) and other databases. We were interested in publications from countries with roughly the same epidemiological profile as Cameroon and where homosexuality is still criminalized. The objective was to make comparisons and draw conclusions that could be applied to Cameroon.

### 2.1. Barriers to accessing traditional HIV testing for MSM

Conventional HIV services done by health care providers at the health facilities level have proven to be a limited strategy for reaching MSM. They face many structural, cultural, and legal barriers in accessing these services.

#### 2.1.1. Structural barriers

##### 2.1.1.1. The number of drop-in center

MSM have easy access to HIV testing through community-based organizations. A good number of DIC offer a “friendly” service, i.e., free of any stigma (11). They have been set up in Cameroon within the framework of the United States’ programs for the fight against AIDS, notably PEPFAR and the Global Fund. They offer a wide range of services for the prevention, testing, psychosocial care, and treatment of HIV. The goal is to allow the beneficiary access to all the services in one place and to relieve hospital congestion (12). Unfortunately, there are very few centers offering these services in Cameroon. They are mainly found in Yaoundé and Douala, the two largest metropolises in Cameroon. The strategy of providing services through DIC is more adapted for key populations (13). Therefore, if we want to reach many MSM, especially those who are “hard to reach”, it is important to set up DIC in the ten regions of Cameroon.

##### 2.1.1.2. Poor training of health providers in stigma-free outreach

Numerous studies have shown that MSM does not attend hospitals and clinics because of the stigma and discrimination (14). Indeed, medical and paramedical staff are often not trained in the stigma-free reception of these populations (15). The health services available are not well adapted to the needs of these populations (16). To date, there are no modules on sexual orientation and gender identity in the training curricula of healthcare professionals. Therefore, they are unaware of the importance of treating patients without judgment (17). Although the Hippocratic Oath (18) recommends treating all patients without distinction, discrimination cases remain legion. Non-Government Organizations working with key populations and sexual and gender minorities in Cameroon has documented a good number of human rights violation done at health facilities based on sexual orientation and gender identity.

#### 2.1.2. Cultural and legal barriers

Many cultural and legal barriers limit access to HIV testing for MSM. The Cameroonian penal code condemns same-sex relationships through Law 347-1 (19). This law is the source of many violations based on sexual orientation and gender identity (20). In addition, the existing DIC is not officially registered as targeting MSM. As a result, many beneficiaries are afraid to go to this DIC for fear of being reported or recognized by their relatives who might suspect them of homosexuality (21).

Moreover, in Cameroon, cultural groups implicitly or explicitly promote discrimination and stigmatization of MSM through their way of thinking, feeling, and acting (22). In addition, some healthcare providers rely on these beliefs to withhold medical treatment from MSM (23). These violations widen the gap between MSM and hospitals (24).

### 2.2. Prerequisites for the implementation of de-medicalized and decentralized HIV

Taking a certain number of measures is crucial to implement the DDHT strategy. Community actors must be trained, and a means must be put in place to quickly link people who test positive for HIV to care through active referral.

#### 2.2.1. The training of peer educators for DDHT implementation

**Figure 1:**
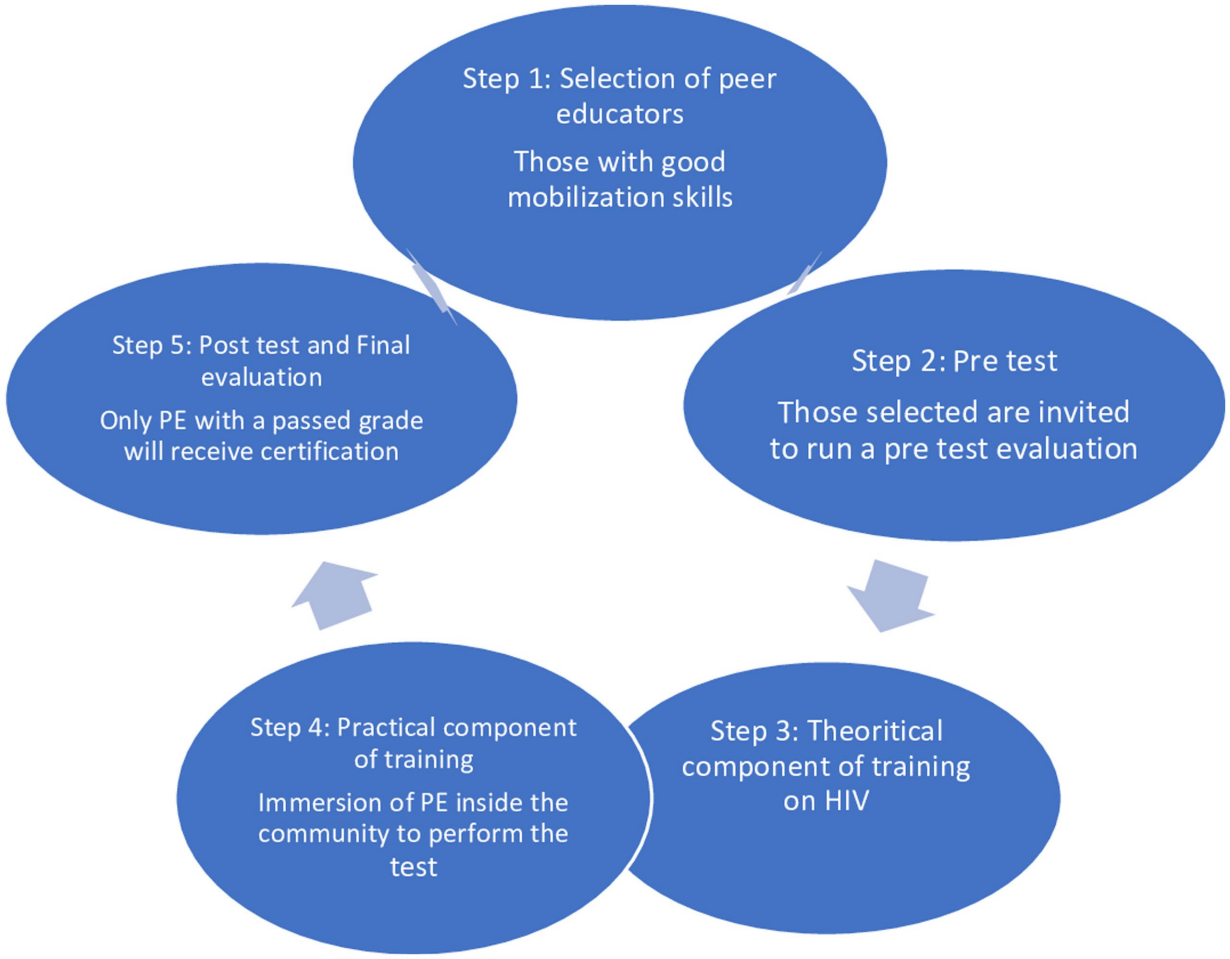
The training cycle of peer educators for DDHT implementation.

Training peer educators is a crucial step to implementing this strategy effectively. It enables them to build their capacities and gives them the legitimacy to perform this medical task (see Figure 1).

The training concerns peer educators selected among the best mobilizers and those with a level of education that allows them to read and write. The first step of this training includes a pre-knowledge test (25). The aim is to evaluate their knowledge of HIV prerequisites and their ability to persuade MSM to get tested. The training should occur over at least three to 5 days, with a theoretical and a practical component. The theoretical component provides knowledge about HIV prevention and testing, risk behaviour assessment, and behaviour change through communication.

The practical component lets peer educators know how to use the rapid HIV testing kit, especially the ALERE Determine™ HIV1/2. It is important to emphasize the interpretation of results according to the national algorithm of Cameroon (26). Using the finger prick technique is vital to limit the risk of blood exposure accidents. Although wearing a gown is not mandatory in implementing this strategy, it is still necessary to insist on wearing gloves and alcohol hand rubs for hand hygiene (27). During this training, it is essential to emphasize role model playing to strengthen the peer educators’ capacities in pre- and post-HIV test counseling. During the training, it is necessary to include a time for immersion inside the community. A post-test knowledge test and a final evaluation are conducted at the end of this strategy. Only successful participants receive certification, which gives them the right to test people; those with a low mark will retake the training during another session and receive accreditation once they have a passed grade.

The case study from Uganda below perfectly illustrates how relevant and practical training of community health workers can improve the uptake of HIV testing and linkage to Antiretroviral treatment.

##### 2.2.1.1. Case study 1

Training of community health extension workers to perform HIV testing and linkage in Uganda (28).

Through this study, community health extension workers (CHEWs) were trained; they attended a five-day training that introduced them to the biology of HIV, the conduct of HIV screening tests, the maintenance of confidentiality regarding HIV testing, HIV prevention messages, principles of biosafety, and linkage, referral, and reporting requirements. The training curriculum followed the Uganda Ministry of Health AIDS Control Program HIV/AIDS awareness training materials. CHEWs were each provided a $30 monthly stipend and a field-testing kit that included a bicycle, field bag, umbrella, gumboots, reporting booklet, pens, HIV testing kits, biohazard bags, lancets, and gloves. Supplies were replenished monthly. At the initiation of the project, Integrated community-based initiatives staff assisted each pair of CHEWs in developing a route for approaching each consecutive household in their administrative parish. Trained CHEWs tested 43,696 persons for HIV infection during the six-month program period. Nine-hundred seventy-four participants (2.2%) were identified as HIV positive, and 623 participants (64%) were linked to HIV care. An estimated 69% of adult residents received testing as part of this campaign. The program costs $3.02 per person test, $135.70 per positive person identified, and $212.15 per HIV-positive person linked to care.

#### 2.2.2. Active referral to treatment for positive cases

To implement DDHT, it is also essential to establish collaborations with HIV treatment centers. So far, in Cameroon, the initiation of antiretroviral therapy (ART) is only done at health facilities. People tested for DDHT must be accompanied to the hospital by community agents through the active referral model (17, 29). Since 2016, Cameroon has applied the “test and treat” strategy, which allows the rapid initiation of treatment for HIV-positive patients (30). If this active referral system is implemented, many people may be lost to follow-up. Indeed, hard-to-reach MSM has little knowledge about HIV and are often likely to enter denial and refuse treatment camp (29). However, experience has shown that community workers can easily convince these people if they are well-trained in counseling (11). Active peer referral also reduces the waiting time for treatment because once the person goes to the hospital, they are automatically put on ARVs for free (17, 31). A retrospective observational study from Zambia will be described in the line below. It will show how peer community health workers have contributed to improving the 3×′95′ targets related to HIV testing, linkage to treatment and viral load suppression.

##### 2.2.2.1. Case study 2

Peer community health workers improve HIV testing and ART linkage among key populations in Zambia: retrospective observational results from the Z-CHECK project, 2019–2020 (32).

It is a study from Zambia with peer community health workers (CHWs); they were trained through the Zambia Community HIV Epidemic Control for Key Populations (Z-CHECK) project. This project aimed to improve HIV case-finding, linkage, and treatment adherence at the community level for key populations (KPs) in Zambia. CHWs offered HIV testing in safe spaces and escorted through active referral newly HIV-diagnosed clients for same-day ART initiation. Z-CHECK provided HIV testing for 9,211 KPs, of whom 2,227 were HIV positive (positivity yield, 24%). Among these, 1901 (85%) were actively linked to Antiretroviral therapy. Program strategies contributing to high positivity yield and linkage included peer KP CHWs, social network testing strategies and opportunities for same-day ART initiation. Challenges to program implementation included stigma and discrimination among HCWs and KP CHW attrition, which may be explained by high mobility.

### 2.3. The added value of de-medicalized and decentralized testing

Considering that many health professionals need to support this strategy thoroughly, it is essential to highlight how relevant and suitable it can be for marginalized and vulnerable populations living in a repressive environment.

#### 2.3.1. Screening a large number of HIV-positive MSM

The results obtained during the implementation of DDHT have shown that this strategy can reach a large number of MSM compared to traditional medical testing strategies (33). In addition, this strategy gets the most at-risk MSM who never have or only minimally accessed testing (33). Therefore, the number of positive cases is high according to the yield result. Furthermore, the technical equipment deployed to implement this strategy is simple and easily transportable. In addition, the community agents trained to implement this strategy do not need to wear white coats that could be mistaken for health care providers. Therefore, they can offer this service in hot spots where MSM meets.

#### 2.3.2. Practical task shifting toward communities

The DDHT strategy transfers skills toward the community as recommended by UNAIDS (34, 35). Community members are no longer only relayed to prevention through peer education. It is now possible that they will also participate in the continuum of care and treatment for people living with HIV. However, it is essential to emphasize that healthcare professionals consider the training offered to peer educators insufficient. They must be equipped to deal with counselling and psychosocial follow-up (35). The study proposes that only psychosocial counselors should be trained to implement this strategy to guarantee all the required standards (16, 25). However, the community-based ARV dispensing strategy puts the most affected communities at the heart of the HIV response. Therefore, it is essential to decentralize HIV treatment through a task-shifting process to reach the 3×′95′ target by 2030 (8).

#### 2.3.3. DDHT: an entry point to improve the sexual and reproductive health of hard-to-reach MSM

DDHT is also a good entry point to prevent the spread of other STIs and to improve the sexual and reproductive health of hard-to-reach MSMs. Today many MSM are still excluded from HIV testing, especially in low and middle-income countries where exposure to HIV risk is high, where MSM are highly stigmatized and therefore are less likely to go for standard testing. Increasing the number of entry points for non-medicalized HIV testing by implementing community-based testing is a public health priority not only to control the HIV epidemic but also to reduce inequalities in access to HIV care.

Studies suggest that MSM who are reached through a community-based approach are those who are most at risk of HIV and other sexually transmitted infections (28). It is also possible to train peer educators or community health workers to perform and interpret other rapid tests such as syphilis and hepatitis B. During the implementation of DDHT, peer educators sensitize hard-to-reach MSMs on how to prevent and treat HIV and other STIs through educational talks, they also provide them with condoms and lubricants. Integrated other sexual and reproductive health services while performing the DDHT is a very effective strategy to reach the 95/95/95 targets (36). In a nutshell, everything is done for risk reduction and to offer stigma-free services to MSM.

#### 2.3.4. Continuous monitoring throughout the 3×′95′ targets

The DDHT approach is also relevant because it facilitates follow-up of people who have tested positive throughout the 95/95/95 continuum of care. In a study conducted in Zambia among transgender women, community workers made active referrals to hospitals for treatment initiation (37). They ran a battery of psychosocial follow-up activities for care retention to achieve an undetectable viral load, which is currently the only way to control the virus (38). Indeed, this community-based approach provides better results in the continuum of HIV prevention, testing and care because it is different and adapted to the needs of the beneficiaries.

#### 2.3.5. Cost-effective strategy

The implementation of DDHT is a cost-effective strategy because it involves community health workers who are less paid than trained health professionals (35). For example, for the implementation of the CHAMP (Continuum of HIV Prevention, testing and treatment with most at-risk populations in Cameroon) project, which was funded by USAID et implemented by Care Cameroon between 2014–2021, CHW trained to implement the DDHT received 45$ per month compared with laboratory staff who received 450$ per month with the same HIV testing indicators. Also, to implement the DDHT strategy, buying technical materials such as centrifuge, EDTA tub, syringes, and needles is optional when the test is performed at health facilities. The DDHT only requires alcohol for hand disinfection and a needle for a finger prick.

### 2.4. Limits of de-medicalized and decentralized HIV testing strategy

Even if we acknowledge that this strategy has many advantages and is suitable for testing hard-to-reach MSM, it is also important to point out its limits, including the breach of confidentiality and the lack of trustfulness it inspires in beneficiaries.

In fact, by involving peer educators in HIV testing, it is essential to be aware of the risk of breaching confidentiality (11).

Doing an HIV test requires a lot of courage and responsibility, so the person doing this test must keep the results confidential. Psychosocial counselors are well-trained to do pre- and post-HIV test counseling. They know well how to assure people who have tested HIV positive, and they know what words to use to convince clients that the results of their HIV test will remain confidential. Unfortunately, peer educators are not sufficiently trained to do this high-standard counseling with beneficiaries. Since they are also community members, they are not trusted by their peers; they are not credible to do it. People are not confident about their HIV because they are afraid that they will disclose their test results to others. An excellent way to deal with this is to strengthen the capacity of peer educators in counseling. They must also sign a form stating that all information they have while doing their work will remain confidential.

Another limitation is the lack of trust in the quality of HIV testing beneficiaries perform. Many people believe that only healthcare workers can provide HIV testing. They have spent much time at school learning their profession, so it is impossible for peer educators who have been trained for a few days to have the same skills. So, people are reluctant to trust the HIV results they received from peer educators. But those with a positive impact are actively referred to a health facility or drop-in center for a confirmatory test done by a health professional. It is impossible to link someone to care using the test done by peer educators; they are just running an orientation test.

### 2.5. Quality assurance: comparison of finger prick versus full blood for ELISA

Increasing the number of people aware of their status is a key issue to end AIDS by 2030. The most important results are the impressive high acceptability of rapid testing and the very high acceptability of HIV testing offered in community-based strategy. People need to trust the test which is being offered. Rapid testing from finger-stick whole blood is more acceptable and feasible than ELISA for routine universal HIV testing. Larger use of rapid tests, ideally free of charge, by general practitioners could reduce the pool of infected patients unaware of their status (39). When it comes to comparing the sensitivity of rapid HIV testing, it is sometimes difficult to know which samples (oral fluid, full blood, or serum) provide good results. Oral fluid is less sensitive than full blood which is in turn less sensitive than serum (40). So, we can conclude that the use of ELISA for HIV which is using serum is more sensitive than the use of finger prick with a full blood sample. But for economic purposes, the use of finger pricks is more acceptable in some countries because it does not require a lot of logistics.

## 3. Conclusion and implication for future research

Our aim in this article was to show that DDHT is appropriate to target “hard-to-reach” MSM living in countries where homosexuality remains criminalized. This mode of testing circumvents structural, legal, and social barriers to accessing health services for these populations. However, medical personnel remain skeptical and distrustful of this approach due to inadequate training. However, it is nonetheless true that, if trained and supervised, community actors could become the foundation for achieving the goal of ending AIDS by 2030. The DDHT also brings the issue of task shifting in comprehensive HIV care back to the forefront. Particular attention should be given to community-based HIV initiation, which until now has been the exclusive domain of the medical profession.

## Data availability statement

The original contributions presented in the study are included in the article/supplementary material, further inquiries can be directed to the corresponding author.

## Author contributions

JO and PN: conception and study design. JO: manuscript drafting. PN, AE, and EK: manuscript revision. PN: guarantor of the study. All authors contributed to the article and approved the submitted version.

## Conflict of interest

The authors declare that the research was conducted in the absence of any commercial or financial relationships that could be construed as a potential conflict of interest.

## Publisher’s note

All claims expressed in this article are solely those of the authors and do not necessarily represent those of their affiliated organizations, or those of the publisher, the editors and the reviewers. Any product that may be evaluated in this article, or claim that may be made by its manufacturer, is not guaranteed or endorsed by the publisher.
